# Competing Endogenous RNA Networks in Glioma

**DOI:** 10.3389/fgene.2021.675498

**Published:** 2021-04-29

**Authors:** Liang Cen, Ruochen Liu, Wei Liu, Qianqian Li, Hongjuan Cui

**Affiliations:** ^1^State Key Laboratory of Silkworm Genome Biology, Southwest University, Chongqing, China; ^2^Cancer Center, Medical Research Institute, Southwest University, Chongqing, China; ^3^Department of Psychology, The Second Affiliated Hospital of Chongqing Medical University, Chongqing, China; ^4^Ministry of Education Key Laboratory of Child Development and Disorders, Department of Neurosurgery, National Clinical Research Center for Child Health and Disorders, Children’s Hospital of Chongqing Medical University, Chongqing, China

**Keywords:** ceRNET, ceRNA, glioma, ncRNA, cancer driver dysregulation, glioblastoma

## Abstract

Gliomas are the most common and malignant primary brain tumors. Various hallmarks of glioma, including sustained proliferation, migration, invasion, heterogeneity, radio- and chemo-resistance, contribute to the dismal prognosis of patients with high-grade glioma. Dysregulation of cancer driver genes is a leading cause for these glioma hallmarks. In recent years, a new mechanism of post-transcriptional gene regulation was proposed, i.e., “competing endogenous RNA (ceRNA).” Long non-coding RNAs, circular RNAs, and transcribed pseudogenes act as ceRNAs to regulate the expression of related genes by sponging the shared microRNAs. Moreover, coding RNA can also exert a regulatory role, independent of its protein coding function, through the ceRNA mechanism. In the latest glioma research, various studies have reported that dysregulation of certain ceRNA regulatory networks (ceRNETs) accounts for the abnormal expression of cancer driver genes and the establishment of glioma hallmarks. These achievements open up new avenues to better understand the hidden aspects of gliomas and provide new biomarkers and potential efficient targets for glioma treatment. In this review, we summarize the existing knowledge about the concept and logic of ceRNET and highlight the emerging roles of some recently found ceRNETs in glioma progression.

## Introduction

Gliomas are the most common and malignant primary brain tumors, accounting for about 30% of all primary brain tumors and 80% of malignant ones (Weller et al., 2015). The origin of gliomas is thought to be from neuroglial stem or progenitor cells. Based on morphological similarities to the neuroglial cells of normal brain, the World Health Organization (WHO) 2007 classification system categorizes gliomas into astrocytomas, oligodendrogliomas, mixed oligoastrocytic gliomas, or ependymomas and into I–IV grades with grades I and II and grades III and IV considered low- and high-grade gliomas, respectively (Louis et al., 2007). Patients with high-grade glioma, such as glioblastoma (GBM, a grade IV astrocytoma), have a median survival time of only 15 months after initial diagnosis (Jemal et al., 2010; Rynkeviciene et al., 2019). Research over the past decade using advanced sequencing technologies has unraveled molecular alterations or biomarkers underlying gliomas, which updated our understanding of glioma’s biology and resulted in a new classification system (Louis et al., 2016) with more precision for gliomas. This system integrated histology and molecular biomarkers, including *IDH* (encoding isocitrate dehydrogenase) mutation and 1p/19q-codeletion status (Louis et al., 2016). Despite these progresses, as well as progress in surgical resection, radiotherapy, and chemotherapy, the prognosis for patients with high-grade gliomas remains dismal (Dong and Cui, 2020). Besides that, some low-grade gliomas can develop into secondary high-grade ones after surgical resection, radiotherapy, or chemotherapy (Hamisch et al., 2017). A better understanding of the molecular mechanisms of gliomagenesis is urgently needed to develop potential new biomarkers and therapeutic strategies for improved treatments.

Dysregulation of oncogenes (e.g., *RAS*, *PIK3CA*, and *MYC*) and/or tumor-suppressive genes (e.g., *PTEN*, *TP53*, and *RB1*) leads to cell transformation (Singh et al., 2002; van’t Veer et al., 2002; Ballestar and Esteller, 2008; Liu et al., 2021). Enormous efforts have been devoted to illustrating the dysregulation mechanisms of these cancer driver genes at the transcriptional and post-transcriptional levels. Noticeably, more than 75% of the human genome can generate RNA transcripts, of which only approximately 2% are messenger RNAs (mRNAs) that contain cancer driver genes, and the majority of the rest are noncoding RNAs including microRNAs (miRNAs), long noncoding RNAs (lncRNAs), transcribed pseudogenes, and circular RNAs (circRNAs) (Hangauer et al., 2013; Abdollahzadeh et al., 2019). These noncoding RNAs did not draw attention until in the recent years, and our understanding of their function is still in infancy and requires more research. In 2011, Salmena et al. (2011) proposed that mRNAs, lnRNAs, and transcribed pseudogenes regulate each other *via* acting as competing endogenous RNAs (ceRNAs) to compete for binding of shared miRNAs. This milestone conception of ceRNA implies that all of the above-mentioned types of RNA transcripts, even protein-coding mRNAs themselves, can perform post-transcriptional regulation and constitute ceRNA regulatory networks (ceRNETs), which may profoundly affect the expression of cancer driver genes and promote tumorigenesis.

Examples of ceRNA crosstalk have been described in the latest research on glioma. Indeed the dysregulation of ceRNETs between different kinds of RNAs contributes to the establishment of the hallmarks of different subtypes of gliomas, suggesting the important roles of ceRNETs in the development of gliomas. Therefore, understanding this novel language of RNA crosstalk will give a new insight into gene regulatory networks, open a new window to better understand the hidden and complex aspects of gliomas, and provide a new way to find specific biomarkers and potential efficient therapeutic targets for the diagnosis and treatment of gliomas. In this review, we first introduce the pieces of knowledge of ceRNA hypothesis, particularly highlighting their building blocks including miRNAs, mRNAs, lncRNAs, circRNAs, and transcribed pseudogenes as well as the logic for effective ceRNA crosstalk. Then, we specifically discuss the latest discoveries of distinct ceRNETs in glioma research.

## Participants and Logic of ceRNA Hypothesis

### miRNAs

miRNAs are small single-stranded RNAs (approximately 22 nucleotides) that play key roles in ceRNA crosstalk. They bind to miRNA response elements (MREs) on target RNAs through sequence complementarity, which reduces the stability of targets or restricts their translation. MREs can be found in 5’ untranslated regions (5’ UTRs), coding sequences, and especially 3’ untranslated regions (3’ UTRs) of various types of RNA transcripts, such as mRNAs, lncRNAs, transcribed pseudogenes, and circRNAs. Most miRNAs pair imperfectly with their targets, and pairing between miRNA (two to eight nucleotides, especially six or seven nucleotides from the 5’ end of miRNA) and 3’ UTR of target mRNA is often crucial. Each miRNA can regulate up to thousands of target RNAs, and miRNAs can act in a combinatorial manner if a target RNA has multiple different MREs. miRNA-mediated regulation is estimated to affect a large proportion of human transcriptome, which makes miRNA an important modulator in numerous diseases, including various types of cancers.

**FIGURE 1:**
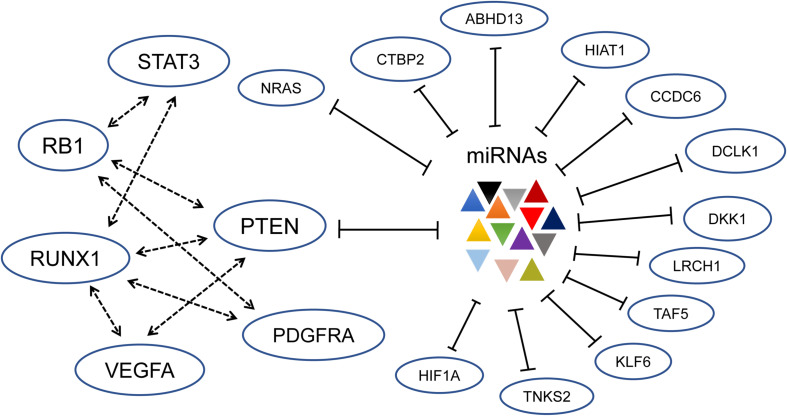
Established driver genes of gliomagenesis constitute a complex ceRNET; especially the ceRNAs for PTEN are shown (Sumazin et al., 2011). Through shared miRNA, the six cancer driver genes including PTEN, RB1, STAT3, PDGFRA, RUNX1, and VEGFA regulate each other. At the same time, other genes constitute a subnetwork and interact with PTEN. The dotted arrows represent activation, and the lines with blunt ends indicate inhibition.

The above-mentioned miRNA → RNAs regulation model has been updated by introducing the concept of ceRNAs. As shown in Figure 1, RNA transcripts that share the same MREs can regulate each other’s expression by competing for the same pool of miRNAs and thus modulating the availability of miRNAs. A new miRNA-mediated regulation model is RNAs ↔ miRNAs ↔ RNAs based on the concept of ceRNA. This means that miRNAs no longer act only as active regulators but that they are also passively regulated by their target RNAs. In addition, even a few miRNAs and related target RNAs can generate a complex ceRNET. Studying the larger interconnected ceRNET, rather than isolated ceRNA pair interactions, may generate deeper insight into ceRNA-mediated gene regulation in a setting that is closer to physiological conditions.

### mRNAs, lncRNAs, circRNAs, and Transcribed Pseudogenes

The central dogma of molecular biology is that information on the DNA is transcribed into mRNAs that, in turn, are used as templates for protein synthesis. There are approximately 20,000 protein-coding genes in the human genome (Baltimore, 2001). Many of them, including cancer driver genes, harbor MREs, making their expression profoundly affected by the cognate miRNAs (Friedman et al., 2009). The finding of ceRNA implies that mRNAs possess a regulatory function independent of protein coding function. As a result, the ceRNA activity of mRNAs may confer them independent and even opposite roles to their encoded protein in a process such as tumorigenesis (Salmena et al., 2011). In this scenario, gross genomic losses or amplifications that commonly happened in cancers could potentially affect the function of ceRNAs in these regions and interrupt the related ceRNET. In addition, gene loss events should be distinguished with point mutations, as the former lose both protein-coding sequence and MREs, while the latter lose protein function but retain ceRNA function. The potential role of mRNA-mediated ceRNET has been confirmed in glioma (Sumazin et al., 2011).

lncRNAs are a large variety of RNA transcripts longer than 200 nucleotides without protein-coding capacity but with a similar structure to mRNAs since they typically have a 5’ m7G cap and 3’ poly (A) tail (Ulitsky and Bartel, 2013). The expression of lncRNAs is more tissue specific and dynamic, suggesting that they have distinct biological roles (Deveson et al., 2017). Dysregulation of cancer-related lncRNAs plays important roles in tumorigenesis, and an increasing amount of lncRNAs has been linked to gliomagenesis (Kiang et al., 2015; Peng et al., 2018; Rynkeviciene et al., 2019). lncRNAs perform their regulatory function through interacting with DNA, mRNAs, other non-coding RNAs, and proteins, covering almost all aspects of gene expression regulation including chromatin modification, transcription, post-transcription, and translation (Wang and Chang, 2011). Functionally, lncRNAs can act as signals, decoys, guides, scaffolds, and sponges. Particularly, the miRNA sponging function of lncRNAs that inhibits miRNAs makes lncRNA an important active player in the ceRNET (Xia et al., 2014; Greco et al., 2019; Wang L. et al., 2019; Ebrahimpour et al., 2021).

CircRNAs are a class of endogenous non-coding RNA without a 5’ m7G cap and 3’ poly (A) tail structure and are formed by the circularization of pre-RNAs *via* back splicing (Li X. et al., 2018; Kristensen et al., 2019). They are widely present in a variety of human cells. Because of lack of exposed 5’ and 3’ ends, circRNAs are more stable than linear RNAs in terms of resistance to degradation by exonucleases or RNase R (Suzuki et al., 2006). Thus, circRNAs are stable in human body fluids, including blood and saliva, making them suitable biomarkers for diagnosis of cancer (Bahn et al., 2015; Memczak et al., 2015). CircRNAs carry out their function through a variety of mechanisms, such as acting as ceRNAs, interacting with RNA-binding proteins, alternative splicing, and translation (Li X. et al., 2018; Kristensen et al., 2019). Due to the high stability of circRNAs, the sponge effect of circRNAs on miRNAs has been appreciated. Rybak-Wolf et al. (2015) revealed that circRNAs are highly abundant in the mammalian brain, dynamically expressed, and conserved among human, mouse, and *Drosophila*. Furthermore, Song et al. (2016) developed a computational tool called UROBORUS to detect circRNAs in total RNA-seq data and found that more than 476 circRNAs were differentially expressed in control brain tissues and gliomas. Increasing recent reports have illustrated the crucial role of dysregulated circRNAs in gliomagenesis, showing great potential as valuable diagnostic and therapeutic biomarkers (Jin et al., 2018; Ding et al., 2020; Long et al., 2020).

Pseudogenes are genomic loci similar to the known genes but lost their protein coding ability as a result of premature stop codons, deletions, insertions, or frameshift mutations (D’Errico et al., 2004). Therefore, they were considered as “non-functional,” “junk,” or “genomic fossils,” until recently their roles in various biological processes have been illustrated. Genomic sequencing analyses showed a huge number of pseudogenes (∼19,000) in humans, and many of them are transcribed and well-conserved (Pink et al., 2011). The ENCODE project further revealed that the transcription of some pseudogenes is tissue specific or constitutive (Pei et al., 2012). Mechanically, transcribed pseudogenes regulate the expression of target genes by the generation of endogenous small interference RNAs (siRNAs) (Watanabe et al., 2008) and antisense transcripts (Zhou et al., 1992) or acting as ceRNAs (Poliseno et al., 2010). Since pseudogenes are highly similar to their ancestral protein-coding genes, they can actively compete for the same pool of miRNAs through shared MREs (An et al., 2017). Poliseno et al. (2010) firstly reported that pseudogene PTENP1 derepresses the expression of tumor-suppressor gene PTEN through competing for PTEN-targeting miRNAs in prostate cancer cells and colon carcinoma cells, therefore exerting a tumor-suppressive role. Furthermore, they extended their analysis to other cancer driver genes with pseudogenes, such as oncogene *KRAS* and its pseudogene *KRAS1P*. Thereafter, the ceRNA function of more pseudogenes is revealed in various cancers, including breast cancer and gliomas (Shi X. et al., 2016; Li et al., 2017b; Wang Y. et al., 2019).

### Logic for Effective ceRNA Crosstalk

The effectiveness and result of crosstalk between ceRNAs are regulated by multiple factors, including relative concentration and subcellular localization of the ceRNAs and miRNAs, number of shared MREs, and miRNA-ceRNA binding affinity (Salmena et al., 2011; Sanchez-Mejias and Tay, 2015). Both mathematical models and experimental models support that ceRNA crosstalk conform to a titration mechanism that is sensitive to the relative abundance of miRNA/target RNAs and often exhibit a threshold-like manner (Buchler and Louis, 2008). Alterations of the ceRNA levels should be large enough to overcome or relieve suppression on competing ceRNAs by the miRNAs. Similarly, absent expression or overexpression of shared miRNAs will abolish ceRNA competition. When the levels of miRNA and ceRNAs are near equimolar, optimal ceRNA crosstalk is expected to happen, in which one ceRNA has the biggest effect on its ceRNA partners (Mullokandov et al., 2012; Bosia et al., 2013). Subcellular localization influences ceRNA’s accessibility to miRNAs. Not all miRNAs are present everywhere and at all times, and many RNA-binding proteins can profoundly affect the localization or compartmentation of RNA transcripts through mechanisms including phase separation (Venables et al., 2009; Fox et al., 2018; Ries et al., 2019). The number of shared miRNAs among ceRNAs is important for effective ceRNA crosstalk; the more shared, the deeper the communication (Ala et al., 2013; Figliuzzi et al., 2013). MREs on ceRNAs are not equal. Although two MREs can bind the same miRNA, their partially different nucleotide composition contributes to the distinct binding affinity between miRNAs and ceRNAs (Salmena et al., 2011). The nonreciprocal competing effect between partially and perfectly complementary ceRNAs was predicted computationally and validated experimentally in cultured human cells using synthetic gene circuits (Yuan et al., 2015). Collectively, the above-mentioned factors should be considered when studying ceRNAs and especially assigning their contribution to specific human diseases, which may facilitate the translation of research results to clinical application.

## Extensive mRNA Crosstalk in Glioma Through ceRNA Mechanism

Sumazin et al. (2011) presented a pioneer and comprehensive study of mRNA–mRNA crosstalk through shared miRNAs in GBM. Using computational tools, an extensive ceRNET, consisting of about 7,000 genes and more than 248,000 miRNAs, is constructed. Further biochemical assays in cell lines confirmed that established drivers of tumor initiation and subtype implementation are indeed regulated by this ceRNET, including PTEN, RB1, STAT3, PDGFRA, RUNX1, and VEGFA (Figure 1). Specifically, they focused on 13 genes, including *ABHD13*, *CCDC6*, *CTBP2*, *NRAS*, and *RB1*, and confirmed that these genes can act through ceRNA mechanism to regulate the expression of PTEN and *vice versa* (Figure 1). The overexpression of PETN 3’ UTR increases the expression of 13 ceRNAs, elevates PTEN protein level, and decreases the growth rates of glioma cells, while knockdown of each of the 13 genes can reduce PTEN 3’ UTR luciferase expression and significantly promote glioma cell growth. The silencing effect mediated by the ceRNA mechanism is comparable to that of siRNA-mediated PTEN silencing. Moreover, PTEN composes a subnetwork with the known drivers of glioma tumorigenesis and GBM subtypes, i.e., RB1, STAT3, PDGFRA, RUNX1, and VEGFA. The ectopic expression of the 3’ UTRs of genes in this subnetwork can upregulate the expression of the other genes. Therefore, the ceRNA mechanism provides a way for these cancer drivers to be coordinately expressed through a shared miRNA pool, which is implicated in high-grade gliomagenesis (Chow et al., 2011).

## Examples of lncRNA–miRNA–mRNA ceRNETs in Glioma

**FIGURE 2:**
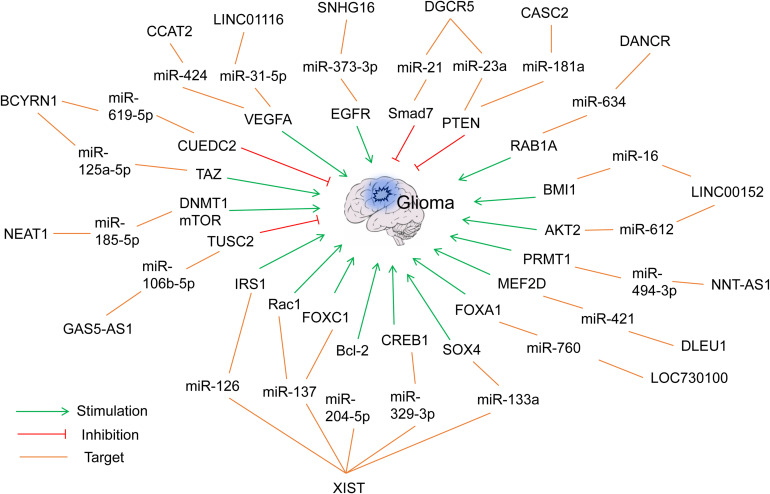
Representative lncRNA-mediated ceRNETs in gliomas.

**FIGURE 3:**
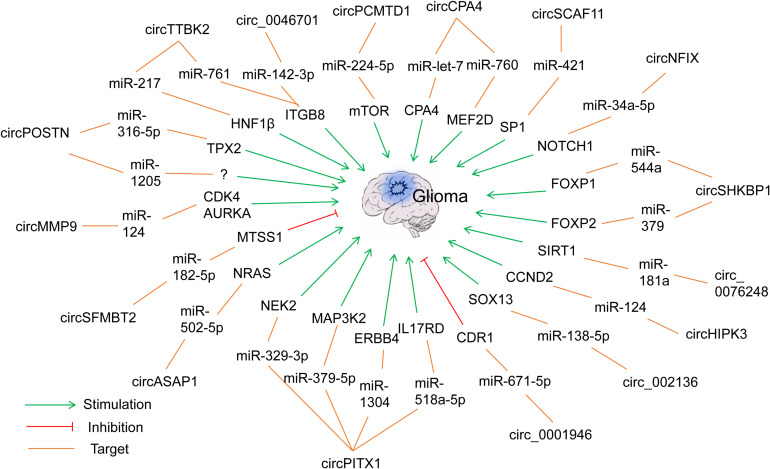
Representative circRNA-mediated ceRNETs in gliomas.

The ceRNET concept is one of the hot research topics in recent years, and reports of lncRNA- or circRNA-mediated ceRNETs in glioma research are increasing fast using computational or experimental methods (Wu and Qian, 2019; Zhu et al., 2020c). For example, Zhu et al. (2020c) constructed a comprehensive lncRNA–miRNA–mRNA ceRNET consisting of 61 lncRNAs, 12 miRNAs, and 92 mRNAs through a computational method. Here we will discuss and highlight several lately discovered ceRNETs in the following sections. More ceRNETs are summarized in Figures 2, 3 and Tables 1, 2.

**TABLE 1:** Representative lncRNA-mediated ceRNETs in glioma.

lncRNA	Competitor (mRNA)	Shared miRNA(s)	ceRNA role	Related glioma hallmark	References
BCYRN1	CUEDC2	miR-619-5p	Tumor suppressive	Proliferation and migration	Mu et al., 2020
	TAZ	miR-125a-5p	Oncogenic	Proliferation, migration, and invasion	Yu et al., 2020
CCAT2	VEGFA	miR-424	Oncogenic	Proliferation, apoptosis, and angiogenesis	Sun S.L. et al., 2020
SNHG16	EGFR	miR-373-3p	Oncogenic	Proliferation, migration, and invasion	Zhou X.Y. et al., 2020
DGCR5	Smad7	miR-21	Tumor suppressive	Proliferation, migration, invasion, and apoptosis	He et al., 2020
	PTEN	miR-23a			
NNT-AS1	PRMT1	miR-494-3p	Oncogenic	Cell viability, proliferation, migration, and invasion	Zheng et al., 2020
GAS5-AS1	TUSC2	miR-106b-5p	Tumor suppressive	Proliferation, migration, and invasion	Huang et al., 2020
LINC01116	VEGFA	miR-31-5p	Oncogenic	Proliferation, migration, invasion, and angiogenesis	Ye et al., 2020
MALAT1	Rap1B	miR-101	Oncogenic	Proliferation and apoptosis	Li et al., 2017c
LINC00152	AKT2	miR-612	Oncogenic	Proliferation, migration, invasion, and colony formation	Cai et al., 2018
	BMI1	miR-16		Proliferation, migration, and invasion	Chen et al., 2018
DANCR	RAB1A	miR-634	Oncogenic	Proliferation and colony formation	Xu D. et al., 2018
DLEU1	MEF2D	miR-421	Oncogenic	Proliferation, migration, invasion, and apoptosis	Feng et al., 2019
LOC730100	FOXA1	miR-760	Oncogenic	Proliferation, migration, invasion, and apoptosis	Li Q. et al., 2019
XIST	SOX4	miR-133a	Oncogenic	Proliferation, invasion, and EMT	Luo et al., 2020
	IRS1	miR-126		Cell viability, migration, invasion, and apoptosis	Cheng et al., 2020
	Rac1	miR-137		Proliferation	Wang et al., 2017
	FOXC1			Angiogenesis	Yu et al., 2017
	Bcl-2	miR-204-5p		Proliferation, migration, invasion, and apoptosis	Shen et al., 2020
	CREB1	miR-329-3p		Proliferation, invasion, apoptosis, and radiosensitivity	Wang Y.P. et al., 2020
CASC2	PTEN	miR-181a	Tumor suppressive	Proliferation and chemoresistance	Liao et al., 2017
CCAT1	FGFR3 and PDGFRα	miR-181b	Oncogenic	Proliferation, migration, EMT, and apoptosis	Cui et al., 2017
DLEU2	PDK3	miR-186-5p	Oncogenic	Colony formation, migration, and invasion	Xie et al., 2019
PSMB8-AS1	DDIT4	miR-22-3p	Oncogenic	Proliferation, apoptosis, and radiosensitivity	Hu et al., 2020
MATN1−AS1	CHD1	miR-200b, miR-200c, and miR-429	Oncogenic	Proliferation and apoptosis	Zhu et al., 2020b
NEAT1	DNMT1 and mTOR	miR-185-5p	Oncogenic	Proliferation, migration, EMT, and apoptosis	Zhu et al., 2020b

**TABLE 2:** Representative circRNA-mediated ceRNETs in glioma.

circRNA	Competitor (mRNA)	Shared miRNA(s)	ceRNA role	Related glioma hallmark	References
circPOSTN	TPX2	miR-361-5p	Oncogenic	Proliferation, cell growth, and apoptosis	Long et al., 2020
	?	miR-1205		Cell growth, migration, invasion, and apoptosis	Yang Y. et al., 2019
circCPA4	CPA4	miR-let-7	Oncogenic	Proliferation and invasion	Peng et al., 2019
	MEF2D	miR-760		Proliferation, migration, invasion, apoptosis, and radiosensitivity	Zhang Y. et al., 2020
circ_0001946	CDR1	miR-671-5p	Tumor suppressive	Proliferation, migration, invasion, and apoptosis	Li and Diao, 2019
circMMP9	CDK4 and AURKA	miR-124	Oncogenic	Proliferation, migration, and invasion	Wang et al., 2018
circASAP1	NRAS	miR-502-5p	Oncogenic	Proliferation, apoptosis, and chemoresistance	Wei et al., 2020
circPITX1	IL17RD	miR-518a-5p	Oncogenic	Proliferation, migration, invasion, and apoptosis	Zhan et al., 2019
	ERBB4	miR-1304		Proliferation, migration, invasion, and apoptosis	Chen M. et al., 2020
	MAP3K2	miR-379-5p		Proliferation and apoptosis	Lv et al., 2019
	NEK2	miR-329-3p		Cell growth, colony formation, and radiosensitivity	Guan et al., 2020
circPCMTD1	mTOR	miR-224-5p	Oncogenic	Cell viability, proliferation, migration, and invasion	Zheng et al., 2019
circSCAF11	SP1	miR-421	Oncogenic	Proliferation and invasion	Meng et al., 2019
circEZH2	DDAH1 and CBX3	miR-1265	Oncogenic	Cell growth, migration, invasion, and apoptosis	Gao et al., 2020
hsa_circ_0000177	FZD7	miR-638	Oncogenic	Proliferation and invasion	Chen and Duan, 2018
circITCH	ITCH	miR-214	Tumor suppressive	Proliferation, migration, and invasion	Li F. et al., 2018
circNFIX	NOTCH1	miR-34a-5p	Oncogenic	Proliferation, migration, and apoptosis	Xu H. et al., 2018
hsa_circ_0007534	ZIC5	miR-761	Oncogenic	Proliferation and migration	Li G.F. et al., 2018
hsa_circ_0046701	ITGB8	miR-142-3p	Oncogenic	Proliferation and invasion	Li G. et al., 2018
circTTBK2		miR-761	Oncogenic	Proliferation, invasion, and ferroptosis	Zhang H.Y. et al., 2020
circCFH	AKT1	miR-149	Oncogenic	Proliferation and colony formation	Bian et al., 2018
circHIPK3	IGF2BP3	miR-654	Oncogenic	Proliferation and invasion	Jin et al., 2018
	CCND2	miR-124		Proliferation, migration, and invasion	Liu Z. et al., 2020
	WEE1	miR-124-3p		Proliferation, invasion, and EMT	Xia et al., 2020
	KIF2A	microRNA-524-5p		Proliferation, invasion, apoptosis, and chemoresistance	Yin and Cui, 2020
circTTBK2	HNF1β	miR-217	Oncogenic	Proliferation, migration, invasion, and apoptosis	Zheng et al., 2017
circSHKBP1	FOXP1	miR-544a	Oncogenic	Proliferation, migration, and angiogenesis	He et al., 2018
	FOXP2	miR-379			
hsa_circ_0088732	RAB3D	miR-661	Oncogenic	Migration, invasion, EMT, and apoptosis	Jin et al., 2020
circPTN	SOX6	miR-122	Oncogenic	Proliferation and apoptosis	Chen C. et al., 2020
circHECTD1	SLC10A7	miR-296-3p	Oncogenic	Proliferation and invasion	Li et al., 2021
circSFMBT2	MTSS1	miR-182-5p	Tumor suppressive	Proliferation, migration, and invasion	Zhang S. et al., 2020
circFANCL	HMGB1	miR-337-3p	Oncogenic	Proliferation and apoptosis	Tao et al., 2020
has_circ_0012129	TGIF2	miR-761	Oncogenic	Cell viability, proliferation, colony formation, migration, invasion, and apoptosis	Xu et al., 2020
circ_0079593	KPNA2	miR-499a-5p	Oncogenic	Proliferation, migration, and apoptosis	Yang et al., 2020
circ_0000215	CXCR2	miR-495-3p	Oncogenic	Proliferation, invasion, EMT, and apoptosis	Mutalifu et al., 2020
circABCB10	FABP5	miR-620	Oncogenic	Proliferation, migration, invasion, and apoptosis	Sun W.Y. et al., 2020
hsa_circ_0076248	SIRT1	miR-181a	Oncogenic	Proliferation, invasion, and chemoresistance	Lei and Huang, 2019
circ_0034642	BATF3	miR-1205	Oncogenic	Proliferation, migratory, invasion, and apoptosis	Yang M. et al., 2019
circU2AF1	NOVA2	miR-7-5p	Oncogenic	Proliferation migration, invasion, and apoptosis	Li G. et al., 2019
circ_002136	SOX13	miR-138-5p	Oncogenic	Migration, invasion, and angiogenesis	He Z. et al., 2019
circDICER1	ZIC4	miR-103a-3p and miR-382-5p	Oncogenic	Proliferation, migration, and angiogenesis	He Q. et al., 2019
hsa_circ_0074362	HOXB7	miR-1236-3p	Oncogenic	Proliferation, migration, and invasion	Duan et al., 2018
circELF2	MUC15	miR-510-5p	Oncogenic	Cell growth, migration, invasion, and apoptosis	Zhang and Xu, 2020
hsa_circ_0000337	MAT2A	miRNA-942-5p	Oncogenic	Proliferation, migration, and invasion	Liu N.Z. et al., 2020
circ_0079586	MDM4	miR-183-5p	Oncogenic	Cell growth, migration, invasion, and apoptosis	Chen J. et al., 2020
circENTPD7	ROS1	miR-101-3p	Oncogenic	Proliferation, migration, and invasion	Zhu et al., 2020a
circEPHB4	SOX10	miR-637	Oncogenic	Stemness, proliferation, and glycolysis	Jin et al., 2021
circTOP2A	SUSD2	miR-346	Oncogenic	Cell viability, migration, invasion, and apoptosis	Sang et al., 2021
circ_101064	PIWIL1	miR-154-5p	Oncogenic	Proliferation, invasion, and migration	Zhou H. et al., 2020
circ_0000020	PIK3CA	miR-142-5p	Oncogenic	Proliferation, migration, and invasion	Wang and Zhu, 2021

*^?^Undefined.*

### lncRNA XIST/Multiple miRNAs/Multiple mRNAs

lncRNA X-inactive specific transcript (XIST), located on chromosome Xq13.2, is frequently diagnosed in various cancers, including gastric cancer, lung cancer, and glioma (Wang Y.P. et al., 2020). Acting as a ceRNA, at least five miRNAs (e.g., miR-133a, miR-126, miR-137, miR-204-5p, and miR-329-3p) have been identified in the XIST-mediated ceRNETs that affect multiple hallmarks of glioma progression, including proliferation, apoptosis, migration, EMT, and angiogenesis (Figure 2 and Table 1).

Wang et al. (2017) reported that XIST promotes gliomagenesis through the XIST/miR-137/Rac1 regulatory axis. Specifically, the expression of XIST and miR-137 is significantly up- and down-regulated in glioma tissues, respectively. Overexpression of XIST promotes the proliferation of glioma cells, which can be reversed by miR-137 overexpression (Wang et al., 2017). Ract1 (Ras-related C3 botulinum toxin substrate1) is a member of the Rho family that belongs to the Ras superfamily of GTPases (Coso et al., 1995). It plays a crucial role in the regulation of proliferation, differentiation, and apoptosis of tumor cells and is abnormally expressed in several cancer types, including non-small cell lung cancer (Zhou et al., 2016), breast cancer (Algayadh et al., 2016), and sarcoma (Manara et al., 2016). Interestingly, through sponging miR-137, XIST regulates glioma angiogenesis by regulating FOXC1 (forkhead box C1) expression (Yu et al., 2017). Yu et al. (2017) found that XIST is upregulated in endothelial cells in a blood–tumor–barrier model *in vitro*. FOXC is a transcription factor of the forkhead box family, and it promotes glioma angiogenesis by activating the expression of CXCR7 [chemokine (C–X–C motif) receptor 7b] (Yu et al., 2017).

In 2020, four groups reported novel XIST-mediated ceRNETs that promote glioma progression, i.e., XIST/miR-133a/SOX4 (Luo et al., 2020), XIST/miR-126/IRS1 (Cheng et al., 2020), XIST/miR-204-5p/Bcl-2 (Shen et al., 2020), and XIST/miR-329-3p/CREB1 (Wang Y.P. et al., 2020). Luo et al. (2020) reported that XIST/miR-133a/SOX4 ceRNET regulates the proliferation, invasion, and EMT of glioma. Sox4 is a member of the Sox (SRY-related HMG-box) family of transcription factors and is involved in cell differentiation and proliferation (Tiwari et al., 2013). Cheng et al. (2020) reported that XIST/miR-126/IRS1 ceRNET regulates cell viability, migration, invasion, glucose metabolism, and resistance to apoptosis in glioma cells. IRS1 (insulin receptor substrate 1) is a key target of the insulin receptor tyrosine kinase involved in hormonal control of metabolism (Shah et al., 2004). Furthermore, Cheng et al. (2020) demonstrated that IRS1 promotes glioma progression by activating the PI3K/AKT pathway. Shen et al. (2020) reported that XIST/miR-204-5p/Bcl-2 ceRNET regulates the proliferation, migration, invasion, and apoptosis of glioma cells. XIST can regulate a variety of apoptosis-related genes, including Bax, caspase 3, caspase 9, and Bcl-2; however, only Bcl-2 had been shown to be a direct target of miR-204-5p and was only tested in their study (Shen et al., 2020). Wang Y.P. et al. (2020) reported that XIST/miR-329-3p/CREB1 ceRNET regulates proliferation, invasion, apoptosis, and radiosensitivity in glioma. CREB1 (cAMP response element binding protein 1) is a member of the leucine zipper with a basic domain (bZip) family of transcription factors and regulates responses to a variety of growth factors and stress signals (Wang et al., 2016). In addition to the above-mentioned studies with known competing endogenous mRNAs for XIST, XIST can also sponge other miRNAs to affect glioma progression, such as miR-152 (Yao et al., 2015), yet the downstream mRNA targets remain unknown.

Collectively, these results clearly indicate that lncRNA XIST plays key roles in gliomagenesis by targeting multiple miRNAs and, in turn, de-represses the expression of multiple cancer driver genes. Therefore, XIST-mediated ceRNETs could be potential diagnostic and prognostic biomarkers and therapeutic targets.

### lncRNA BCYRN1/miR-619-5p/mRNA CUEDC2, lncRNA BCYRN1/miR-125a-5p/TAZ

Mu et al. (2020) identified 183 lncRNAs that were significantly differentially expressed in the glioma samples of patients compared with normal control and further investigated the function of lncRNA BCYRN1 (brain cytoplasmic RNA 1, also called BC200) which was the most downregulated one. Functionally, they showed that BCYRN1 overexpression can repress the proliferation and migration of glioma cells, while its knockdown has opposite effects. Mechanically, BCYRN1 acts as a ceRNA to impede gliomagenesis by sponging miR-619-5p to regulate the expression of CUEDC2 (CUE domain-containing protein 2) and PTEN/AKT/p21 pathway (Mu et al., 2020). CUEDC2 is an adapter protein with a CUE domain that is involved in regulating protein stability *via* ubiquitination of specific substrates (Shih et al., 2003). The PTEN/AKT/p21 pathway acts on downstream targets of CUEDC2 to mediate the anti-tumor effect of BCYRN1 (Mu et al., 2020).

In contrast, Liu et al. (2015) showed that BCYRN1 is significantly downregulated during genotoxic stress-induced necrosis in human glioma cell lines, implying an oncogenic function of lncRNA. In 2020, the same group reported that BCYRN1 functions as an oncogene and promotes proliferation, invasion, and migration (Yu et al., 2020). Mechanically, BCYRN1 sponges endogenous tumor suppressor miR-125a-5p to de-depress the expression of TAZ (transcriptional coactivator with PDZ-binding motif). TAZ has been shown to regulate mesenchymal differentiation in GBM, i.e., TAZ is required for self-renewal, invasion, and tumor formation of mesenchymal glioma stem cells (Bhat et al., 2011). Future studies could reconcile the difference between these studies and shed light on the exact role of BCYRN1-mediated ceRNETs in glioma progression.

## Examples of circRNA–miRNA–mRNA ceRNETs in Glioma

### CircPOSTN/miR-361-5p/mRNA TPX2

Long et al. (2020) demonstrated in glioma cells that circPOSTN (has_circ_0030018), as a ceRNA, is involved in the stimulation of cell growth and aerobic glycolysis and inhibition of apoptosis by upregulating the mRNA TPX2 (targeting protein for *Xenopus* kinesin-like protein 2) through sponging miR-361-5p (Figure 3 and Table 2). CircPOSTN (has_circ_0030018), located at chr13:38136718–38161065 (2,656 nucleotides), was screened by high-throughput circRNA microarray to be upregulated in glioma tissues compared with normal tissues. A high level of circPOSTN was significantly associated with larger tumor size, higher WHO grades, and shorter overall survival (Yang Y. et al., 2019). In the study of Long et al. (2020), the effect of circPOSTN on apoptosis, proliferation, and aerobic glycolysis is mitigated by silencing miR-361-5p. miR-361-5p is a tumor suppressor in multiple types of cancers, including prostate cancer (Liu et al., 2014), cutaneous squamous cell carcinoma (Kanitz et al., 2012), hepatocellular carcinoma (Sun et al., 2016a), non-small cell lung cancer (Chen et al., 2016), and breast cancer (Cao et al., 2016). Zhang et al. (2017) reported that miR-361-5p inhibits the migration, invasion, and epithelial–mesenchymal transition of glioma cells *via* regulating the Twist1/Bmi-1 signaling axis. Whether Twist1 is targeted by circPOSTN remains unknown, while Long et al. (2020) found that TPX2 acts as a downstream target of miR-361-5p/circPOSTN in glioma cells. Depletion of circPOSTN or TPX2 significantly suppresses cell proliferation and aerobic glycolysis while promoting the apoptosis of glioma cells (Long et al., 2020). TPX2 is a cell cycle-regulated nuclear protein that functions in proliferation and mitotic spindle assembly (Heidebrecht et al., 1997; Kufer et al., 2002). As an oncogene, TPX2 is involved in multiple cancers, including gastric cancer (Tomii et al., 2017), colon cancer (Wei et al., 2013), lung squamous cell carcinoma (Ma et al., 2006), pancreatic cancer (Ludwig et al., 2017), and prostate cancer (Zou et al., 2018). In glioma cells, TPX2 promotes cell proliferation and invasion by activating the AKT signaling pathway (Gu et al., 2016). On the other hand, Yang Y. et al. (2019) showed that CircPOSTN promotes cell growth and invasion by sponging miR-1205, yet the targets of miR-1205 have not been explored. Therefore, these results illustrate that CircPOSTN play a crucial role in the progression and invasion of gliomas as a miRNA sponger and may be a useful new prognostic biomarker and therapeutic target for gliomas.

### circCPA4/miR-let-7/mRNA CPA4, circCPA4/miR-760/mRNA MEF2D

Hsa_circ_0082374 was screened by Peng et al. (2019) in a circRNA microarray analysis of glioma and matched normal brain tissues as the most up-regulated one among the top 20 up-regulated circRNAs. It locates at chr7:129948146–129964020 and is named circCPA4 as it was assumed to be derived from carboxypeptidaseA4 (CPA4) according to the human reference genome (GRCh47/hg19). A high level of circCPA4 correlates with a poor prognosis of glioma, and the knockdown of it impedes cell proliferation and invasion in glioma. Mechanically, circCPA4 acts as a ceRNA and sponges miR-let-7 to derepress the expression of CPA4 (Peng et al., 2019). CPA4 is a member of the metallocarboxypeptidase family and may be involved in the regulation of peptide hormone activity and hormone-regulated cell proliferation and differentiation (Huang et al., 1999; Tanco et al., 2010). The expression of CPA4 is elevated in multiple types of cancer tissues of patients, such as gastric cancer (Sun et al., 2016b), pancreatic cancer (Sun et al., 2015), breast cancer (Handa et al., 2019), lung cancer (Sun et al., 2016c), and esophageal squamous cell carcinoma (Sun L. et al., 2017), and can be used as a potential diagnostic and prognostic biomarker as well as a therapeutic target.

Zhang Y. et al. (2020) further explored the circCPA4 function in glioma cells and found that suppression of the circRNA inhibits tumor cell proliferation, migration, and invasion while promoting cell apoptosis and radiosensitivity *in vitro* and repressing tumor growth *in vivo*. They pointed out that a higher expression of circCPA4 is positively correlated with tumor size, WHO grade, and poor prognosis in patients. Differently from Peng et al. (2019) in the mechanism, Zhang Y. et al. (2020) revealed that circCPA4 sponges MEF2D (myocyte enhancer factor 2D)-targeting miR-760 to promote glioma progress. Knockdown of miR-760 can reverse the antitumor effects mediated by the suppression of circCPA4. MiR-760 is a well-identified tumor-suppressive miRNA that functions in many types of cancers, including hepatocellular carcinoma (Tian et al., 2018), breast cancer (Han et al., 2016), and non-small cell lung cancer (Zhu et al., 2019), *via* regulating the malignant properties of tumor, such as cell proliferation, apoptosis, migration, and drug resistance. Then, Zhang Y. et al. (2020) confirmed that MEF2D is targeted by miR-760 glioma cells. MEF2D is a transcription factor of the myocyte-specific enhancer factor 2 (MEF2) family involved in the regulation of the differentiation and development of muscle and neuronal cells (McKinsey et al., 2002). Interestingly, miR-760 and MEF2D are also involved in lncRNA LOC730100- and lncRNA DLEU1-mediated ceRNETs in glioma cells, respectively (Feng et al., 2019; Li Q. et al., 2019), implying the potential crosstalk between the circCPA4 and the lncRNAs. These results indicate the importance and complexity of circCPA4-mediated ceRNETs in gliomagenesis, providing potential biomarkers and targets for glioma treatment.

### circ_0001946/miR-671-5p/mRNA CDR1

Circular RNA circ_0001946 (also known as CDR1as and CiRS-7) that derives from chrX:139865339–139866824 is involved in the progression of multiple cancer types, such as esophageal squamous cell cancer (Fan et al., 2019), colorectal cancer (Deng et al., 2020), lung adenocarcinoma (Yao et al., 2019), and glioblastoma (GBM) (Li and Diao, 2019). Li and Diao (2019) revealed that circ_0001946 suppresses GBM progression by activating the expression of CDR1 through sponging miR-671-5p. Circ_0001946 and its competing mRNA CDR1 can inhibit the proliferation, migration, and invasion and promote the apoptosis of GBM cells, while miR-671-5p has the opposite effect. Microarray analyses showed that circ_0001946 and CDR1 were down-regulated in GBM, while miR-671-5 was up-regulated. The genomic region containing miR-671-5p gene is frequently amplified in GBM (Barbagallo et al., 2016), and its regulatory role on circ_0001946 and CDR1 expression was proven by Hansen et al. (2011) earlier using HEK293 cells derived from human embryonic kidney cells. CDR1 (cerebellar degeneration-related autoantigen 1) is encoded by the *CDR34* gene and is required for neuronal–glial functions. Inhibition of CDR1 expression leads to the loss of differentiation of neural cells and neoplastic transformation (Chen et al., 1990; Satoh and Yamamura, 2004). These results suggest that stimulating the circ_0001946/miR-671-5p/CDR1 axis may be a potential therapeutic strategy for GBM treatment.

### circMMP9/miR-124/mRNAs CDK4 and AURKA

CircMMP9 (hsa_circ_0001162) was screened as the circRNA with the greatest differential expression in the GBM tissues compared with the adjacent normal brain tissues in a microarray analysis performed by Wang et al. (2018). It is derived from exons 12 and 13 of MMP9 (matrix metalloproteinase-9), with 328 nucleotides in length. Overexpression of circMMP9 promotes the proliferation, migration, and invasion of GBM cells through sponging miR-124 (Wang et al., 2018). Thereafter, the oncogenic effect of circMMP9 was demonstrated in osteosarcoma (Pan et al., 2019) and oral squamous cell carcinoma (Xia et al., 2019). Cyclin-dependent kinase 4 (CDK4) and aurora kinase A (AURKA) are two downstream targets of miR-124/circMMP9 in GBM cells (Wang et al., 2018). Furthermore, Wang et al. (2018) found that eukaryotic initiation factor 4A3 (EIF4A3) binds to the MMP9 mRNA transcript to induce circMMP9 cyclization, which improves the circMMP9 level in GBM. EIF4A3 is a component of the exon junction complex involved in exon splicing (Chan et al., 2004). The expression of EIF4A3 shows prognostic significance in Chinese Glioma Genome Atlas but not The Cancer Genome Atlas (TCGA) database, which may be caused by the difference in sample size and ethnicity between the two data sets (Wei et al., 2020).

### circPITX1/Multiple miRNAs/Multiple mRNAs

circPITX1 (hsa-circ-0074026) is another circRNA found to be up-regulated in GBM tissues compared with the noncancerous controls in the microarray analysis performed by Wang et al. (2018). It locates in chr5:134363423–134365011, with 2,383 bp in length. Recently, four groups reported different circPITX1-mediated ceRNETs in glioma cells, i.e., circPITX1/miR-518a-5p/IL17RD (Zhan et al., 2019), circPITX1/miR-1304/ERBB4 (Chen M. et al., 2020), circPITX1/miR-379-5p/mitogen-activated protein kinase 2 (MAP3K2) (Lv et al., 2019), and circPITX1/miR-329-3p/NIMA-related kinase 2 (NEK2) (Guan et al., 2020).

Zhan et al. (2019) further confirmed that circPITX1 is upregulated in cancerous tissues of 52 patients and four glioma cell lines, which is correlated with the patient’s tumor size and WHO grade. Through gain- and loss-of-function assays, they demonstrated that circPITX1 can promote the growth, migration, invasion, and survival of glioma cells. Mechanically, Zhan et al. (2019) proposed that circPITX1 promotes IL17RD (interleukin 17 receptor D) expression by sponging miR-518a-5p. miR-518a-5p also plays a tumor-suppressive role in colorectal cancer (Rubie et al., 2014), diffuse large B cell lymphoma (Huang et al., 2021), and gastrointestinal stromal tumor (Shi Y. et al., 2016) but an oncogenic role in ovarian cancer (Zhang N. et al., 2020). IL17RD can interact with the IL-17 receptor and mediates IL-17 signaling (Rong et al., 2009). The oncogenic role of IL17RD has been shown in colon cancer (Pekow et al., 2017) and colorectal cancer (Yu et al., 2019).

Similar to the study of Zhan et al. (2019); Chen M. et al. (2020) observed a clinical significance of circPITX1 in larger tumor size and higher WHO grade of patients and an inhibitory effect of circPITX1 knockdown on the proliferation, migration, invasion, and survival of glioma cells. Mechanically, they proposed that circPITX1 regulates ERBB4 expression to promote glioma progression by sponging miR-1304. ERBB4 (HER4) belongs to the epidermal growth factor (EGF)/ERBB family of receptor tyrosine kinases, which also includes the EGF receptor (EGFR/HER1/ERBB1), ERBB2 (HER2/Neu), and ERBB3 (HER3) (Qiu et al., 2008). The abnormal expression of each ERBB is associated with many human cancers (Hynes and Lane, 2005).

Lv et al. (2019) performed circPITX1 knockdown experiments and observed reduced proliferation and increased apoptosis of GBM cells. Mechanically, they proposed that circPITX1 promotes GBM progression by sponging miR-379-5p to increase the expression of MAP3K2 (Lv et al., 2019). The effects of circPITX1 knockdown on the proliferation and apoptosis of GBM cells can be rescued partly by upregulating MAP3K2. MAP3K2 is a member of the serine/threonine protein kinase family (Mazur et al., 2014). It preferentially activates other kinases of the MAP kinase signaling pathway and is frequently overexpressed in multiple human cancers including non-small cell lung cancer (Yu et al., 2015), hepatocellular carcinoma (Shi et al., 2020), cutaneous melanoma (Chen et al., 2019), and breast cancer (Wu et al., 2017).

Guan et al. (2020) reported that the down-regulation of circPITX1 leads to reduced viability, glycolysis, colony formation, and radioresistance of glioma cells *in vitro* and suppresses tumor growth *in vivo*. Mechanically, Guan et al. (2020) proposed that circPITX1 promotes NEK2 expression by sponging miR-329-3p. Depletion of miR-329-3p reverses the inhibitory effects of circPITX1 knockdown on glycolysis and radioresistance. It has been shown that miR-329-3p plays a tumor-suppressive role in multiple cancers, including non-small cell lung cancer, osteosarcoma, cervical cancer, and hepatocellular carcinoma, through regulating the proliferation and migration of tumor cells (Chang et al., 2017). Furthermore, Guan et al. (2020) demonstrate that NEK2 acts downstream of miR-329-3p/circPITX1 to affect the glycolysis and radiosensitivity of glioma cells. NEK2 is a conserved centrosome kinase of the NIMA-related kinase family, with abnormal expression in a wide variety of human cancers (Hayward and Fry, 2006). NEK2 is widely upregulated in gliomas and associated with WHO grades, proliferation, and prognosis in malignant gliomas (Liu et al., 2017). An earlier study by Ye et al. (2018) showed that NEK2 is overexpressed in the glioma tissues and is targeted by miR-128 involved in regulating the apoptosis of glioma cells.

Collectively, these studies solidly confirmed the crucial roles of circPITX1-mediated ceRNETs in the regulation of multiple hallmarks of gliomas, including proliferation, migration, invasion, apoptosis, and treatment resistance. Considering that circPITX1 knockdown or overexpression has no side effect on the cell proliferation and apoptosis of normal human astrocytes (Chen M. et al., 2020), targeting circPITX1 may be a valuable and promising strategy for glioma treatment.

## Pseudogene-Mediated ceRNETs in Glioma

The finding of pseudogene-mediated ceRNETs laid the foundation for the establishment of the ceRNA hypothesis (Poliseno et al., 2010; Salmena et al., 2011), yet the description of this type of ceRNETs in glioma is less than that of lncRNA- and circRNA-mediated ceRNETs. PTEN pseudogene-1 (PTENP1), the pseudogene of PTEN, acts through ceRNA mechanism to inhibit cancer progression in prostate cancer (Poliseno et al., 2010) and breast cancer (Gao et al., 2019). Although PTENP1 is also found to be involved in regulating the proliferation and invasion of glioma cells, whether its anti-tumor effect is mediated by ceRNA mechanism has not been reported (Hu et al., 2019).

### ANXA2P1

Three annexin A2 pseudogenes, including ANXA2P1, ANXA2P2, and ANXA2P3, are significantly upregulated, along with their parent gene annexin A2 (ANXA2), which is correlated with poor survival outcome of glioma patients (Li et al., 2017b). Whether the co-expression of the pseudogenes (ANXA2P1, ANXA2P2, and ANXA2P) and parent gene (ANXA2) is caused by shared miRNA(s) or ceRNET(s) remain unknown. Yu et al. (2019) identified five pseudogenes correlating with glioma survival from the TCGA dataset and established a complex ceRNET consisting of three pseudogenes (ANXA2P2, EEF1A1P9, and FER1L4), 72 microRNAs, and 322 targeted genes (Wang Y. et al., 2019). However, the computational ceRNET and each ceRNA pair need further experimental validation. Du et al. (2020) reported an experimentally validated ceRNET involving ANXA2P2, in which ANXA2P2 functions as a ceRNA to regulate the expression of lactate dehydrogenase A (LDHA) by sponging miR-9 in GBM (Du et al., 2020). The ANXA2P2/miR-9/LDHA ceRNET regulates glucose metabolism, proliferation, and apoptosis of GBM (Du et al., 2020). LDHA catalyzes the final step of aerobic glycolysis and is abnormally expressed in many human cancers (Li et al., 2017a).

### PDIA3P1

Wang S. et al. (2020) reported a new pseudogene-mediated ceRNET in glioma, protein disulfide isomerase family A member 3 pseudogene 1 (PDIA3P1)/miR-124-3p/RELA. PDIA3P1 is a 2,099-bp fragment mapped to chromosome 1q21.1. High PDIA3P1 expression is correlated with EMT, extracellular matrix disassembly, and angiogenesis and can promote the migration and invasion of glioma cells (Wang S. et al., 2020). Mechanically, PDIA3P1 sponges miR-124-3p to upregulate RELA expression and, in turn, activates the downstream NF-κB pathway, which promotes a highly invasive mesenchymal (MES) transition of glioma cells (Wang S. et al., 2020). RELA (v-rel avian reticuloendotheliosis viral oncogene homolog A) gene encodes the major component of the NF-κB complex (Hashimoto et al., 2011). Intriguingly, hypoxia inducible factor 1 (HIF1) upregulates the transcription of PDIA3P1 by directly binding its promoter, linking hypoxia to MES transition (Wang S. et al., 2020). In addition, the ceRNA function of PDIA3P1 has also been reported in oral squamous cell carcinoma (Sun C.C. et al., 2017) as well as its oncogenic effect in hepatocellular carcinoma (Kong et al., 2017). These results underscore the importance of PDIA3P1-mediated ceRNET in cancer progression and suggest it as a promising prognostic biomarker and therapeutic target in glioma treatment.

## Conclusion

Despite the improved surgical resection, radiotherapy, and chemotherapy, the prognosis for patients with high-grade gliomas is still poor. Understanding the molecular mechanisms underlying gliomagenesis is urgently needed to “break the ice.” Formally proposed in 2011 (Salmena et al., 2011), the ceRNA hypothesis opens up new avenues for basic cancer research, including glioma (Di Palo et al., 2020; Liu Z. et al., 2020; Wang S. et al., 2020). Research in the past decade have shed light on the dysregulated ceRNETs consisting of coding and non-coding RNAs (miRNAs, lncRNAs, circRNAs, and transcribed pseudogenes) in gliomas. As we have discussed here, various hallmarks of gliomas, such as cell proliferation, growth, invasion, EMT, apoptosis, angiogenesis, chemo-resistance, and radio-resistance, are associated with dysregulated ceRNETs. Illustration of the different nature of the RNA interaction within the ceRNETs will provide new insights into the initiation and progression of gliomas and therefore novel biomarkers for the diagnosis, prognosis, and targets for glioma treatment.

## Author Contributions

LC wrote the manuscript. RL drew the cartoon figures. WL collected the articles. HC and QL provided the idea and revised the manuscript. All authors contributed to the article and approved the submitted version.

## Conflict of Interest

The authors declare that the research was conducted in the absence of any commercial or financial relationships that could be construed as a potential conflict of interest.
